# Transmural Migration of a Retained Surgical Sponge Causing Contained Ileal Perforation

**DOI:** 10.7759/cureus.101826

**Published:** 2026-01-19

**Authors:** Sridhar V Prabhu, Vivek Lanka, Srikanth Marthandam, Ravi Teja Chettubattina, Shanmathi Palanimuthu

**Affiliations:** 1 Department of Radiology, All India Institute of Medical Sciences, Mangalagiri, Guntur, IND; 2 Department of General Surgery, All India Institute of Medical Sciences, Mangalagiri, Guntur, IND

**Keywords:** computed tomography abdomen, foreign body reaction, gossypiboma, imaging, intestinal perforation, postoperative complications, retained surgical sponges, textiloma, transmural migration

## Abstract

Retained surgical sponges are rare postoperative complications that may remain clinically silent or present late with nonspecific symptoms. Transmural migration into the bowel is an uncommon sequela, resulting from a chronic inflammatory reaction and gradual erosion of the bowel wall, and may manifest as obstruction or contained perforation. We report a case of a 54-year-old woman who presented three months after a total abdominal hysterectomy with persistent post-surgical abdominal pain, vomiting, weight loss, and recent-onset constipation. Contrast-enhanced computed tomography (CT) demonstrated a circumferential mural thickening of a distal ileal loop with a serpiginous hyperdense structure corresponding to a radiopaque marker, internal mottled gas lucencies producing a spongiform appearance, intramural air foci, adjacent extraluminal air, and surrounding mesenteric fat stranding, consistent with a contained ileal perforation due to the transmural migration of a retained surgical sponge. Surgical resection with primary anastomosis confirmed the diagnosis. This case highlights characteristic CT features and underscores the critical role of imaging in the timely recognition of this rare postoperative complication.

## Introduction

Retained surgical sponges, also referred to as gossypibomas or textilomas, are rare but potentially serious postoperative complications. Despite advances in surgical safety protocols, such events continue to be reported and may remain clinically silent for prolonged periods due to underreporting and delayed symptom onset [1]. Patients often present months to years after surgery with nonspecific symptoms such as abdominal pain, constipation, obstruction, or perforation, which may obscure the underlying diagnosis [2].

The transmural migration of a retained surgical sponge into the gastrointestinal tract is an uncommon sequela that occurs secondary to a chronic inflammatory response and gradual erosion of the bowel wall. This process may culminate in partial or complete intraluminal migration with varied clinical manifestations. Computed tomography (CT) is the imaging modality of choice in delayed presentations, as it reliably demonstrates characteristic features that facilitate accurate diagnosis and guide appropriate management [3]. Herein, we report a rare case of the transmural migration of a retained surgical sponge presenting as a contained ileal perforation three months after abdominal surgery, emphasizing the key CT findings that enabled accurate preoperative diagnosis.

## Case presentation

A 54-year-old woman presented to the emergency department with persistent post-surgical abdominal pain for three months, with recent worsening over the preceding three days. The pain was intermittent and colicky, lasting approximately 10 minutes per episode, and partially relieved with medications. It was associated with vomiting (approximately four episodes per day) over the same duration and recent-onset constipation. She also reported loss of appetite and significant unintentional weight loss of approximately 15 kg over three months. There was no history of abdominal distension, hematemesis, melena, hematochezia, fever, or trauma.

Her medical history was significant for type 2 diabetes mellitus on oral hypoglycemic agents, hypothyroidism on levothyroxine 50 µg, and a cerebrovascular accident one year prior. She had undergone an open total abdominal hysterectomy three months earlier.

On examination, the patient was conscious, coherent, and oriented to time, place, and person. She was hemodynamically stable with a blood pressure of 140/82 mmHg, pulse rate of 88 beats/minute, respiratory rate of 18/minute, and oxygen saturation of 98% on room air. The abdomen was soft, non-tender, and non-distended, with no guarding or rigidity. No palpable mass or organomegaly was noted, and bowel sounds were present. A gaping lower abdominal transverse surgical scar was observed. Systemic examination was otherwise unremarkable, with normal cardiovascular and respiratory findings and no focal neurological deficits.

Laboratory investigations revealed marked leukocytosis with a total leukocyte count of 29.25 × 10³/µL and predominant neutrophilia (90%; absolute neutrophil count: 26.32 × 10³/µL). Hemoglobin was 9.48 g/dL, with a mildly elevated red cell distribution width. Serum procalcitonin was elevated at 3.44 ng/mL, suggestive of an underlying inflammatory or infectious process. A plain radiograph of the abdomen showed no air under the diaphragm or air-fluid levels.

**Figure 1:**
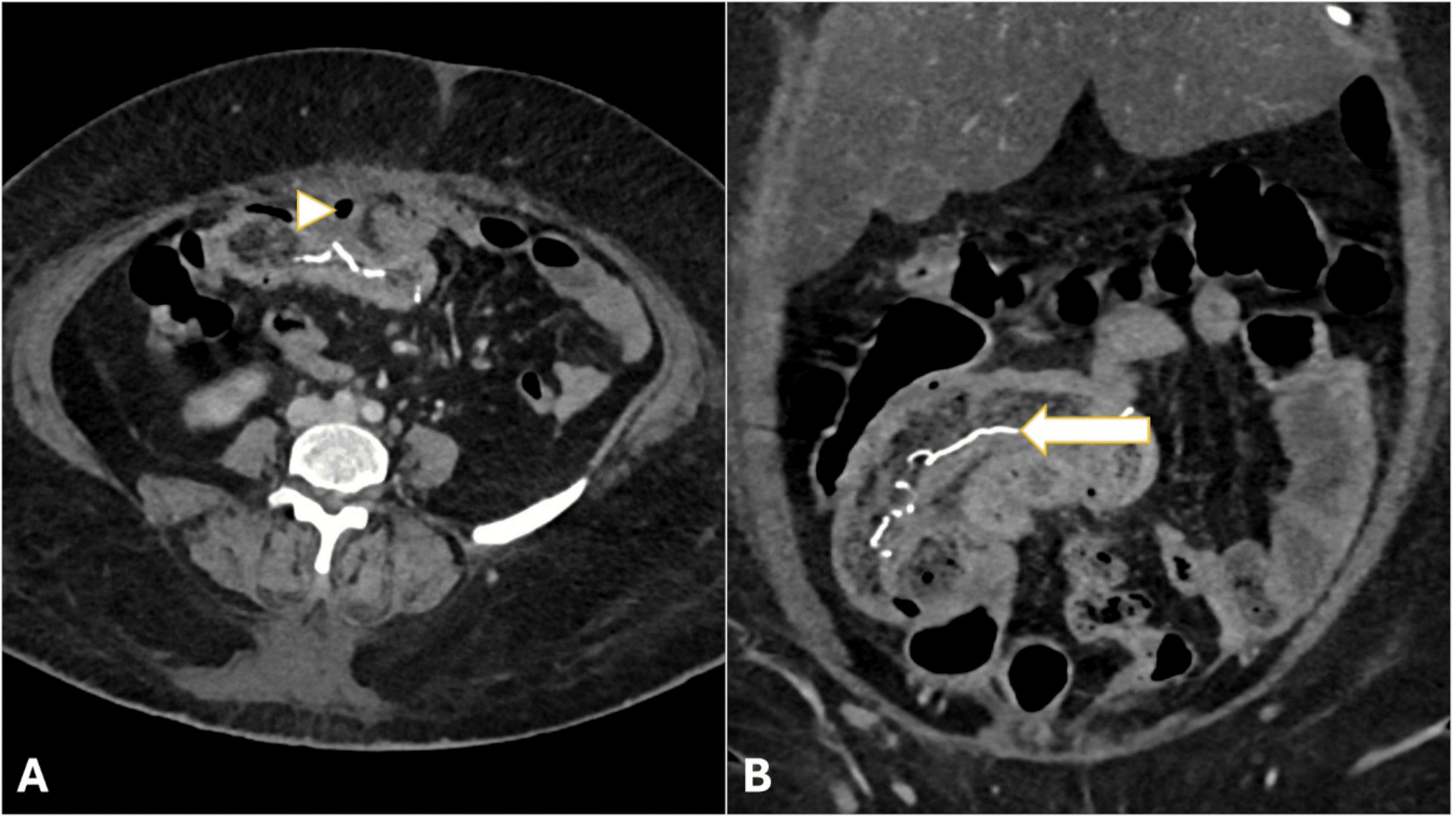
Contrast-enhanced CT images showing a circumferential mural thickening of the distal ileal loop with internal mottled gas lucencies. (A) Axial image: adjacent extraluminal air foci are indicated by the arrowhead. (B) Coronal reformatted image: a serpiginous hyperdense structure, likely representing the radiopaque marker of the retained surgical sponge, is indicated by the arrow. Surrounding mesenteric fat stranding is also noted, consistent with a contained perforation. CT: computed tomography

A contrast-enhanced computed tomography of the abdomen demonstrated a diffuse circumferential mural thickening of a distal ileal loop, with a long serpiginous hyperdense structure (mean Hounsfield unit {HU}: ~688) within the bowel lumen, corresponding to the radiopaque marker of a retained surgical sponge. Multiple intramural air foci and internal mottled gas lucencies imparted a characteristic spongiform appearance. Adjacent extraluminal air foci, mild free fluid, and extensive mesenteric fat stranding were noted, consistent with a contained distal ileal perforation (Figure 1).

In view of the recent surgical history, laboratory evidence of inflammation, and characteristic imaging findings, a diagnosis of contained ileal perforation due to the transmural migration of a retained surgical sponge was made.

**Figure 2:**
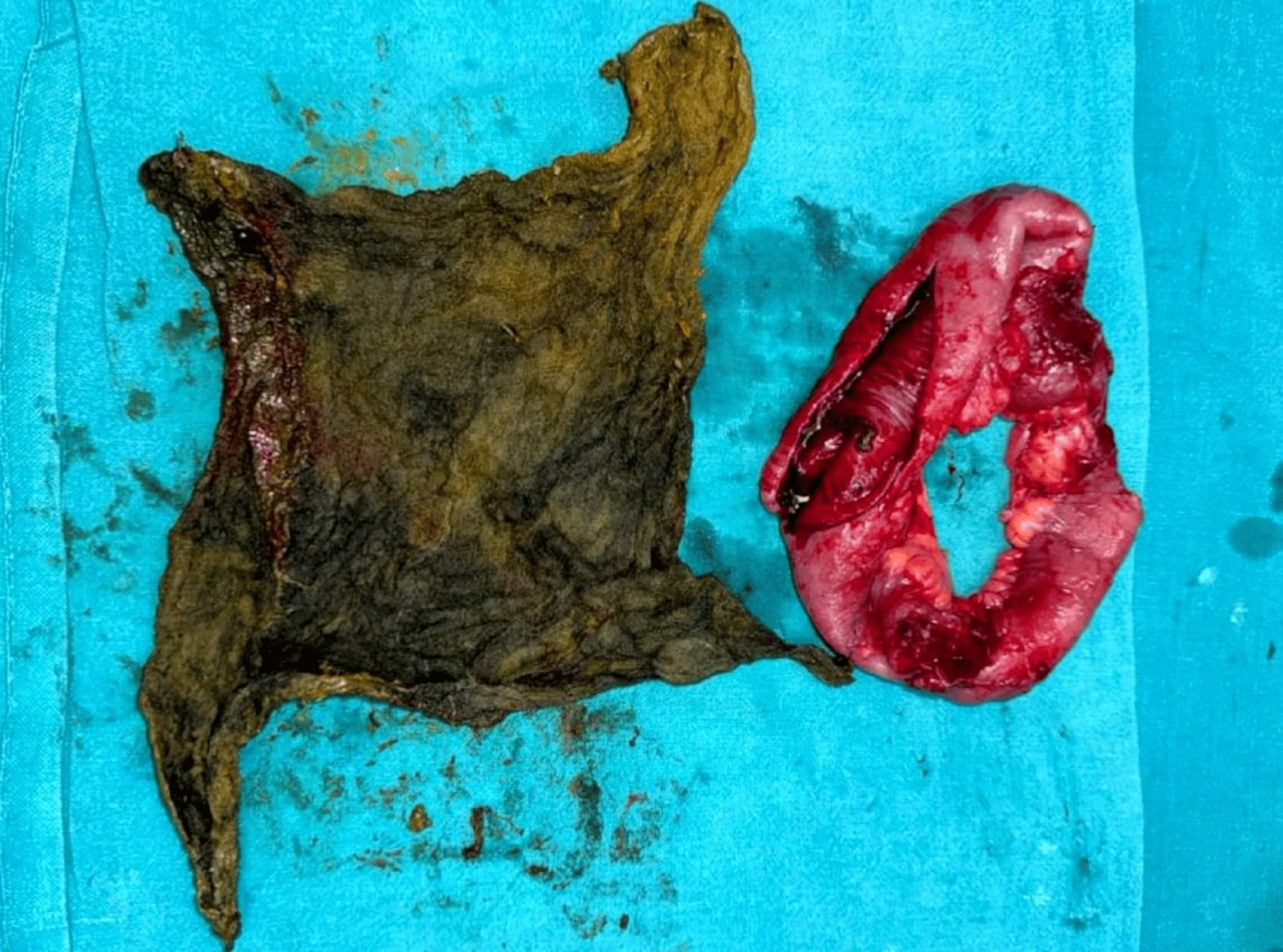
Postoperative gross specimen photograph showing the resected distal ileal segment (approximately 20 cm in length) and the retrieved retained surgical sponge (approximately 10 × 10 inches) placed side by side, confirming the transmural migration of the retained foreign body.

The patient underwent emergency exploratory laparotomy. Intraoperatively, dense adhesions were noted between the ileal loops, sigmoid colon, and the anterior abdominal wall. A 0.5 × 0.5 cm perforation was identified in the distal ileum approximately 20 cm proximal to the ileocecal junction, sealed by surrounding inflammatory adhesions, correlating with the contained perforation seen on CT. A 20 cm segment of the distal ileum containing the perforation was resected, and a primary ileoileal anastomosis was performed. On opening the resected specimen, a retained surgical sponge was identified within the bowel lumen, confirming the radiological diagnosis (Figure 2).

The postoperative course was uneventful. A postoperative ultrasound performed on postoperative day 8 showed no intra-abdominal collection. The patient had a prolonged but stable postoperative recovery and was discharged in a hemodynamically stable condition on postoperative day 26. At one-month follow-up, she remained asymptomatic with no recurrence of abdominal complaints.

## Discussion

Retained surgical sponges provoke a foreign body reaction that may follow different pathological pathways depending on host response and duration. Persistent inflammation can lead to pressure necrosis and eventual erosion into adjacent bowel, allowing the migration of the foreign body into the gastrointestinal lumen [4]. While this process is typically slow and may take years to manifest, the present case demonstrates a relatively short latency period of three months between surgery and symptom onset. Most reported cases of gossypiboma present months to years after the index surgery, although intraluminal migration with early clinical manifestations has been described, suggesting that the rate of migration may vary depending on the inflammatory response, bowel proximity, and local adhesions [5].

The small bowel is the most commonly involved segment in cases of intraluminal migration, likely due to its mobility and proximity to operative fields during abdominal and pelvic surgeries [5]. Partial intraluminal migration may result in localized inflammation or contained perforation, whereas complete luminal migration can lead to intestinal obstruction. In the present patient, the retained surgical sponge was entirely within the distal ileal lumen, resulting in a contained perforation.

Contrast-enhanced computed tomography plays a central role in diagnosing retained surgical sponges, particularly when clinical suspicion is low [6]. Typical CT features include a spongiform mass with internal mottled gas lucencies and a linear hyperdense structure corresponding to the radiopaque marker of the sponge. Associated findings may include bowel wall thickening, mesenteric fat stranding, free fluid, or localized extraluminal air. Other entities may mimic a spongiform appearance on CT, including complex abscesses with gas-forming organisms, bezoars, fecalomas, or necrotic tumors; differentiation relies on the careful assessment of the hyperdense linear marker, surgical history, and the distribution of intraluminal gas. The recognition of this combination of features allows accurate preoperative diagnosis and guides timely surgical intervention.

The clinical spectrum of intraluminal migration is wide. Retained sponges can remain asymptomatic despite advanced migration into the bowel lumen [7]. Conversely, progression to complete luminal obstruction may necessitate urgent surgical management [8]. Although small bowel involvement predominates, migration into other segments of the gastrointestinal tract, including the stomach, has been reported [9]. In selected patients without perforation or peritonitis, minimally invasive retrieval, including endoscopic approaches, has been successful [10].

The awareness of this rare postoperative complication, combined with the recognition of characteristic imaging features, is essential for timely diagnosis and management, reducing morbidity and potential medicolegal consequences. Preventive measures are paramount, including strict adherence to surgical safety protocols such as the WHO Surgical Safety Checklist, meticulous sponge and instrument counts, and the use of radiopaque markers on all surgical textiles. The implementation of these strategies remains the most effective approach to avoid gossypibomas and their associated complications [11].

## Conclusions

The transmural migration of a retained surgical sponge, though rare, is a clinically significant postoperative complication. The recognition of specific CT features, including the spongiform pattern with internal mottled gas lucencies and a linear serpiginous hyperdense structure representing the radiopaque marker, is crucial for early diagnosis. Identifying these signs in patients with prior abdominal surgery and nonspecific symptoms allows for timely surgical intervention, preventing catastrophic complications such as generalized peritonitis and reducing morbidity.
